# Relapse Prevention Group Therapy in Indonesia Involving Peers via Videoconferencing for Substance Use Disorder: Development and Feasibility Study

**DOI:** 10.2196/50452

**Published:** 2024-06-18

**Authors:** Kristiana Siste, Youdiil Ophinni, Enjeline Hanafi, Chika Yamada, Reza Novalino, Albert P Limawan, Evania Beatrice, Vania Rafelia, Peter Alison, Toshihiko Matsumoto, Ryota Sakamoto

**Affiliations:** 1 Department of Psychiatry Faculty of Medicine Universitas Indonesia – Dr. Cipto Mangunkusumo General Hospital Jakarta Indonesia; 2 The Hakubi Center for Advanced Research Kyoto University Kyoto Japan; 3 Department of Environmental Coexistence Center for Southeast Asian Studies Kyoto University Kyoto Japan; 4 Ragon Institute of Mass General, MIT, and Harvard Cambridge, MA United States; 5 Department of Drug Dependence Research National Institute of Mental Health National Center of Neurology and Psychiatry Tokyo Japan; 6 Karisma Foundation Jakarta Indonesia

**Keywords:** substance use disorder, cognitive behavioral therapy, telemedicine, peer involvement, Indonesia, substance use disorders, digital intervention, COVID-19, psychotherapy, drug, mobile phone

## Abstract

**Background:**

Substance use disorder (SUD) is a major health issue in Indonesia, where several barriers to treatment exist, including inaccessibility to treatment services, stigma, and criminalization of drug issues. Peer involvement and the use of telemedicine to deliver psychotherapy are promising approaches to overcome these barriers.

**Objective:**

This study aims (1) to describe the development of a new group psychotherapy coprovided by a health care worker and a peer and (2) to evaluate the acceptability, practicality, and preliminary outcomes of the program delivered via videoconferencing in Indonesia.

**Methods:**

Building upon an established relapse prevention therapy in Japan, we developed a 3-month weekly group therapy module in the Indonesian language. Adjustments were made via focus group discussions with local stakeholders in terms of substance types, understandability, inclusive language, and cultural relevance. A pilot study was conducted to test the new module provided by a peer and a psychiatrist via videoconferencing, termed tele-Indonesia Drug Addiction Relapse Prevention Program (tele-Indo-DARPP), with a pre- and postcontrolled design. We analyzed data from semistructured feedback interviews and outcome measurements, including the number of days using substances and quality of life, and compared the intervention (tele-Indo-DARPP added to treatment as usual [TAU]) and control (TAU only) arms.

**Results:**

In total, 8 people diagnosed with SUD participated in the pilot study with a mean age of 37 (SD 12.8) years. All were men, and 7 (88%) used sedatives as the primary substance. Collectively, they attended 44 of the 48 tele-Indo-DARPP sessions. A total of 3 out of 4 (75%) preferred telemedicine rather than in-person therapy. Positive acceptability and practicality were shown from qualitative feedback, in which the participants who joined the tele-Indo-DARPP reported that they liked the convenience of joining from home and that they were able to open up about personal matters, received helpful advice from peers, and received support from other participants. Providers reported that they feel the module was provider-friendly, and the session was convenient to join without diminishing rapport-building. Meanwhile, troubles with the internet connection and difficulty in comprehending some terminology in the workbook were reported. The intervention arm showed better improvements in psychological health and anxiety symptoms.

**Conclusions:**

Group psychotherapy via videoconferencing coprovided by health care workers and peers was acceptable and practical for participants with SUD and service providers in this study. A large-scale study is warranted to examine the effectiveness of the newly developed module in Indonesia.

## Introduction

Substance use disorder (SUD), defined as the uncontrollable use of psychoactive substances that disrupt daily living, remains a significant health problem worldwide. The burden is especially high in low- and middle-income countries (LMICs), where the absolute mortality due to SUD is higher than in high-income countries [1], and the number of affected individuals exceeds the capacity of accessible, formal care services [2]. This treatment gap seems especially pronounced in Indonesia, the third most populated LMIC worldwide. In 2019, the proportion of people who currently use drugs in Indonesia was estimated to be 1.8%, with marijuana (68%) and amphetamine-type stimulants (ATS, 42%) as the most used substances and unprescribed benzodiazepines and new psychoactive substances (NPS) on a recently increasing trend [3,4]. While the actual SUD prevalence remains unreported, based on the ratio used in global estimates—35 million SUD cases out of 269 million people using drugs [5]—it is estimated that nearly 430,000 Indonesians have SUD. In contrast, there is a severe paucity of available mental health care provisions in the country. Even according to the most recent data, the rate of psychiatrists and mental health nurses was only 0.32 and 2.52 per 100,000 residents, respectively [6]. There is poor personnel decentralization outside of Java island, where the country’s capital is located, and poor facility distribution throughout the country’s 32 provinces, with 7 of them not having a mental hospital [7]. Only a third of all public hospitals and a fifth of all primary health care centers (Puskesmas) nationwide provide mental care services [7,8]. In the Puskesmas, methadone maintenance therapy is available, but implementation issues persist, such as low coverage (5% in 2012) [9], short-lived adherence (61% after 6 months) [10], high prevalence of psychiatric problems, and lower quality of life [11]. Methadone itself is strictly controlled and recipients need to come to Puskesmas nearly every day to take their doses.

Although the existence of standardized therapy for SUD could substantially help close the treatment gap in Indonesia, currently there is none. Here, we would like to propose cognitive behavioral therapy (CBT) as a feasible and scalable therapeutic method for SUD. CBT approaches include motivational interviewing by discussing clients’ ambivalence and efforts to rectify it, as well as relapse prevention by identifying personal triggers and safe methods to quell cravings. The clinical efficacy of CBT in treating SUD has been proven in numerous large-scale studies and meta-analyses [12-14]. In LMICs, 5 randomized clinical trials (RCTs) testing CBT for drug use disorders have been conducted so far [15-19]. Only 2 of them were outpatient-based, 1 in China for opiate dependence [18] and 1 in Iran for methamphetamine use [19], both of which reported positive outcomes in reducing the frequency of use. The provision of CBT is low cost, does not necessitate mental health care specialists, and can be delivered in group therapy, which could thus counterbalance the numerical discrepancy between SUD care providers and recipients. In particular, we would like to elevate the role of peer counselors—people with lived experiences of SUD and recovery who now provide experience-based knowledge as part of the counseling for clients in need—as key CBT providers in formal service care settings. Involvement of peers (also referred to as peer recovery support or involvement of recovery coaches) in SUD care has been shown to be effective in increasing abstinence rate and duration [20-22], increasing outreach in harm reduction programs and efficacy to suppress transmissible infections [23,24], and alleviating societal stigma to drug use [25]. However, based on a recent meta-analysis [26], empirical evidence in LMICs is much more limited. Only 4 studies have been reported so far on the goal of abstinence, including an RCT in Iran [27], a cohort study in Vietnam [28], a pilot mixed method study in Malaysia [29], and a feasibility study in Indonesia, which tested a family-based educational approach for adolescents with SUD delivered by peers [30].

Finally, a telemedicine platform is assumed to further enhance the accessibility and reach of the CBT program. Internet communication would overcome the geographical barriers of the Indonesian archipelago, being beneficial not only in connecting intercities, that is, facilitating connections between providers in central areas and clients in peripheral regions (or vice-versa), but also in connecting intracities, for instance by cutting transportation time in traffic-heavy metropolitan areas, such as Jakarta, or allowing more flexibility in scheduling multiple clients in a group. Privacy is also better protected as clients can receive therapy remotely from their residences, whereas visiting clinics or Puskesmas in person may reveal their SUD diagnosis—a heavily stigmatized health condition—to their social environment. While therapeutic approaches with text messages or web-based applications have been developed for relapse prevention [31], synchronous telemedicine via live videoconferencing may improve rapport, including client engagement with providers and group cohesion, which may all facilitate adherence to therapy. Although videoconferencing has been shown to be effective in delivering psychotherapy for SUD [32-37], recent reviews [38-43] have highlighted 2 important knowledge gaps. First, existing intervention reports have only addressed alcohol and opioid use [32,35-37]. Second, there have been no studies conducted in LMICs that evaluate psychotherapy for SUD delivered via videoconferencing. The number of internet users has been rapidly growing in LMICs including Indonesia. Over 74% of the Indonesian population used smartphones in 2019, a number that is estimated to reach 89% in the next 3 years [44]. Moreover, teletherapy has become an essential approach ever since the COVID-19 pandemic [45]. Its accessibility and effectiveness, even among people with SUD [46,47], may sustain telemedicine as the postpandemic “new normal” for SUD management [48].

Considering the current challenges and opportunities, it would be beneficial to develop a therapeutic program in Indonesia for people with SUD where peers and conventional health care workers work together to deliver the program through videoconferencing. This paper aims (1) to describe the development of a new group psychotherapy for SUD provided by a health care worker and a peer; and (2) to evaluate the acceptability, practicality, and preliminary outcomes of the program delivered via videoconferencing in Indonesia.

## Methods

**Figure 1:**
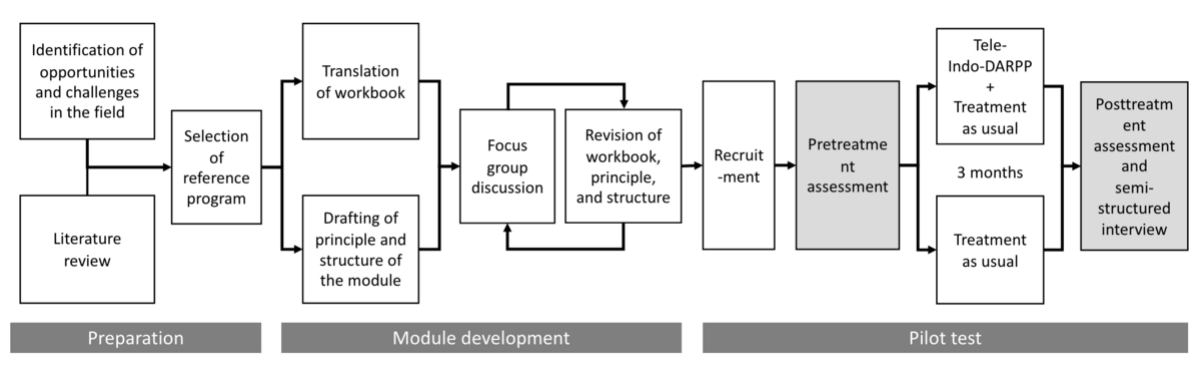
Flowchart describing the processes undertaken in this study.

Our course of action in this study can be divided into 2 phases: module development and pilot study (Figure 1). Tele-Indo-DARPP: tele-Indonesia Drug Addiction Relapse Prevention Program

### Module Development

#### Reference Program

The reference program, the Serigaya Methamphetamine Relapse Prevention Program (SMARPP), and the reasons for choosing it to base our new module were briefly explained previously [49]. Besides its efficacy and scalability, SMARPP has been shown to reduce the stigma held by health care workers [50], as SMARPP is designed to be coprovided by a health care worker and a peer. Thus, the positive interaction between the health care worker and peer might have improved the attitude of health care workers toward people with SUD.

#### Translation and Workbook Development

We first translated the SMARPP module into the Indonesian language. Indonesian addiction psychiatrists reviewed and revised the module to better reflect the local contexts. The module was then named the Indonesia Drug Addiction Relapse Prevention Program (Indo-DARPP). We then held focus group discussions with Indonesian peer counselors (n=6), general practitioners (n=4), psychiatrists (n=3), addiction psychiatrists (n=2), and Japanese researchers (n=2) to further revise the module. All of the members had extensive experience ranging from 2 to 20 years in the addiction field.

Focus discussions led to various adjustments to make the contents of the workbook more locally relevant. Modifications were made to the substances discussed in the module, with the addition of opioids, especially heroin and opioid substitution therapy; the discussions on marijuana and benzodiazepines were extended; the details on antialcohol medications, which are unavailable in Indonesia, were reduced; and the types of prescribed medicines, NPS, and illegal alcohol were adjusted to better reflect popular substances locally. These changes increased the amount of content from 24 chapters or 151 pages in SMARPP to 26 chapters or 200 pages in Indo-DARPP. The content can be divided into 4 separate themes: basic principles of recovery, for example, the definition of addiction, triggers, cravings, and stages of change (chapters 1-8), effects of substances on physical and mental health and the characteristics of each substance (chapters 9-18), and finally, social relationships, relapse cycle, and its prevention (chapters 19-26). Sample pages of the module book can be viewed in Multimedia Appendix 1. The module text is formatted in large 14-point fonts with spacious paragraphs to increase readability and accompanied by both schematic and decorative illustrations to enhance comprehensibility and appeal. A total of three elements were blended (Figure S1 in Multimedia Appendix 1) as follows: (1) user information, which comprises basic information, medical details, evidence-based data, and practical facts, (2) self-assessment queries, which are specific or multiple-choice questions to invite readers in recognizing their situations, and (3) motivational interview, which are open-ended questions to help readers analyze themselves deeper and explore toward a solution and increased motivation. Fonts were enlarged and illustrations were inserted to almost all pages to increase readability, and “take-home messages” were added every 2 to 3 chapters. Colloquial street expressions were incorporated, and stigmatizing terms were replaced with inclusive language, as advised by peer counselors, for example, “people with substance use disorder” instead of “people with addiction,” where the latter has a negative connotation in Indonesian.

The adapted workbook also incorporated cultural concepts. For instance, Ramadan and Christmas were named as long holidays with an increased risk of substance use. Another example is that *wudhu*—the Islamic practice for body purification before prayers—was introduced as a means to cope with cravings for drug use. Information regarding first-aid response to overdose cases was also added. In contrast, cultural nuances from Japan were omitted such as the gambling device *pachinko* and the widespread selling of alcoholic beverages in convenience stores and vending machines.

#### Tele-Indo-DARPP Sessions

The basic group structure, session phases, 12-week schedule, and the tele-delivery method via web videoconferencing (hence tele-Indo-DARPP [tele-Indonesia Drug Addiction Relapse Prevention Program]) were explained previously [49]. Importantly, efforts were made to increase the autonomy of clients and create a safe and comfortable space within the sessions. This was reflected in the main principles that were created specifically for Indo-DARPP and written on the first page of the workbook, namely that (1) clients can freely join regardless of their substance use status; (2) providers should guarantee clients’ safety and comfort (physical wellness, emotional comfort, and privacy) so that they can tell the truth; (3) providers should not judge or negatively react to participants even when they have been using substances; and (4) providers should not put heavy expectations or force behavioral changes on clients. These principles were read aloud by providers at the beginning of each tele-Indo-DARPP session in front of the clients. In contrast, the clients were asked to recite promises to refrain from (1) sharing private information of other clients to any external parties (except by obligation of law or trial); (2) taking photographs or recording audio or video during sessions without permission of everyone pictured; (3) bringing substances into or showing them during the sessions; (4) using substances during the sessions; (5) conducting transaction of substances or handing or receiving substances to or from others; (6) sharing information relating to the session such as schedule, URL address, or meeting access password, among others, to any external parties without permission of the providers; and (7) harassing others, offending others’ identity (gender, race, tribe, and religion), inciting violence or threatening for any reason other clients, providers, or any parties, both during sessions and out of sessions.

#### Providers

One of the most important features of Indo-DARPP is that the sessions were provided by a peer counselor and a health care worker side-by-side, each with their specific roles [49]. Such psychotherapy coprovisioning is a novel concept in Indonesia, especially in the presence of peer counselors who have experience with SUD themselves. While health care workers mainly oversaw the sessions, peer counselors were expected to actively share their own experiences related to the sessions.

### Pilot Study

#### Design and Setting

We conducted a pilot study, using a nonrandomized controlled before-and-after design. The location was Cipto Mangunkusumo General Hospital, the university hospital of Universitas Indonesia, which is located in Central Jakarta, Jakarta province.

#### Recruitment

Convenience sampling was done at the outpatient psychiatric clinic. The inclusion criteria were the following: (1) age between 18 and 65 years, (2) diagnosed with drug or alcohol use disorders as per the *Diagnostic and Statistical Manual of Mental Disorders,* fifth edition, (3) have used primary drugs at least once in the past year, (4) have a device capable of video calling with internet access, and (5) proficient in Indonesian. Exclusion criteria were the following: (1) have a severe physical or mental disability that hinders informed consent or data collection or (2) use inpatient or residential services. The minimal sample size was set at 8, referring to previous online intervention pilot studies [51,52]. We approached 9 participants for recruitment and all of them provided informed consent. One retracted his consent prior to the completion of the pretreatment assessment.

#### Treatment

Participants were allocated to either the intervention or the control arm. Participants in the intervention arm received tele-Indo-DARPP in addition to treatment as usual (TAU), while the control arm received TAU only. The providers of tele-Indo-DARPP were 1 psychiatrist from the Department of Psychiatry at the University of Indonesia and 1 peer counselor from a peer-run nongovernmental organization providing services for people with SUDs. TAU varied among participants and included symptomatic pharmacotherapy (eg, anxiolytics and antidepressants), opioid substitution therapy with methadone, and brief individual psychotherapy with a treating psychiatrist if they are undergoing any treatment with a psychiatrist. The psychotherapy is conducted monthly at most, with each session lasting up to 15 minutes. Structured psychotherapy, such as motivational enhancement therapy or relapse prevention, was not provided to the TAU participants, and there were no group therapy sessions held either. While the psychotherapy uses a motivational interviewing approach in cases of relapse or nonimprovement, the sessions were relatively unstructured, without using any established treatment module for SUD, as there are currently no standardized guidelines available for SUD treatment in Indonesia.

#### Data Collection

Semistructured interviews were conducted after the end of treatment to inquire about the acceptability and practicality of tele-Indo-DARPP and audio recordings were made with the participants’ permission. Structured interviews were also conducted to assess participant characteristics and outcome measurements before and after the treatment provided.

#### Feasibility Measurement

This study defines feasibility as to whether the intervention, tele-Indo-DARPP, can be shaped to be relevant and appropriate to our purpose and sustainable to be tested further in a larger clinical trial [49]. We show the feasibility of tele-Indo-DARPP by quantitatively measuring relevant outcomes and qualitatively examining the feedback from participants after undergoing 12 intervention sessions. We can further identify that the study results fulfill 5 out of 8 areas of focus generally defined in a feasibility study in the context of public health research [53], namely acceptability—how the intended recipients, which in this case are people with SUD, react to tele-Indo-DARPP; demand—viewed from the actual attendance of participants in tele-Indo-DARPP sessions; practicality—the extent to which it is practical for participants and the service providers to join tele-Indo-DARPP sessions; adaptation—focusing on the adaptation to the main changes brought by the tele-Indo-DARPP compared with prior modalities, which are (1) the use of a structured workbook module in group therapy sessions, and (2) the use of video teleconferencing to hold the session and deliver psychotherapy; implementation—whether tele-Indo-DARPP service can be implemented as planned by service providers; integration—the experience of tele-Indo-DARPP implementation vis-à-vis the providers’ familiarity with counseling or psychotherapy service provision; and limited-efficacy testing—comparison of outcome measurements at pre- and posttreatment between the tele-Indo-DARPP+TAU and the TAU-only arms.

The focus on expansion is irrelevant to this study, as tele-Indo-DARPP is not an expansion from a prior psychotherapeutic modality for SUD available in Indonesia.

#### Outcomes

The outcomes, measurements, and corresponding hypotheses are listed in Table 1. The timeline followback was assessed for 30 days [54]. Second, health-related quality of life was measured by the World Health Organization Quality of Life Brief Version (WHOQOL-BREF) [55,56]. The WHOQOL-BREF has 4 domains: physical health, psychological health, social relationships, and environment. Using a standard formula, each domain yields a continuous score that ranges from 0 (impaired health) to 100 (full health). This study hypothesized that the tele-Indo-DARPP arm would show a greater increase in WHOQOL-BREF domain scores than the TAU-only arm. Other outcomes included the Addiction Severity Index [57], University of Rhode Island Change Assessment [58], Brief-Coping Orientation to Problems Experienced (Brief COPE) [59], Symptom Checklist-90 Revised [60], Rey Auditory Verbal Learning Test [61], and internalized stigma of mental illness [62].

While we acknowledge confounding factors potentially affecting the outcomes, such as pharmacotherapy, treatment of comorbidities, and social factors, the current pilot design would not be able to adequately address them [63]. Therefore, we refrain from making any inferences on the efficacy of tele-Indo-DARPP for now and aim to eliminate confounders in a definitive RCT to be done soon [49].

**Table 1:** Outcomes and measurements.

Outcome		Measurement	Data for analysis	Type and score range	Hypothesis for intervention (vs control)
**Primary outcome**			
	Primary substance use	TLFB^a^ for the past 30 days	Number of days using primary substance	Continuous, 0 (no use) to 30 (used every day).	Lower
**Secondary outcomes**					
	Quality of life	WHOQOL-BREF^b^	4 domains: physical health, psychological health, social relationships, and environment, each calculated using a standard formula.	Continuous, 0 (impaired health) to 100 (full health)	Higher
	Addiction severity	ASI^c^	7 composite scores: medical, employment, alcohol use, drug use, legal, family or social, and psychiatric status. Each composite score is calculated using a standard formula.	Continuous, 0 (no problems) to 1 (severe problems)	Lower
	Motivation to change	URICA^d^	Action stage subscale, the sum of 8 items.	Continuous, 8 (not active in behavioral change) to 40 (highly active in behavioral change).	Higher
	Coping strategies	Brief COPE^e^	1 subscale: substance use coping. The sum of 2 items.	Continuous, 0 (not using) to 10 (frequently using)	Lower
	Psychiatric symptoms	SCL-90-R^f^	GSI^g^, an average of 90 items.	Continuous, 0 (no symptoms) to 4 (severe symptoms)	Lower
	Cognitive function	RAVLT^h^	3 test results; immediate, learning, and recalling.	Continuous, 0 (low functioning) to 15 (high functioning)	Higher
	Internalized stigma	ISMI^i^	Total score and 5 subscales: alienation, stereotype endorsement, perceived discrimination, social withdrawal, and stigma resistance (reverse score), an average of 29 items (for total score), 6, 7, 5, 6, and 5 items, respectively (for subscales)	Continuous, 0 (low internalized stigma) to 4 (high internalized stigma)	Lower

^a^TLFB: timeline followback.

^b^WHOQOL-BREF: World Health Organization Quality of Life Brief Version.

^c^ASI: Addiction Severity Index.

^d^URICA: University of Rhode Island Change Assessment.

^e^Brief COPE: Brief-Coping Orientation to Problems Experienced.

^f^SCL-90-R: Symptom Checklist-90 Revised.

^g^GSI: Global Severity Index.

^h^RAVLT: Rey Auditory Verbal Learning Test.

^i^ISMI: Internalized Stigma of Mental Illness.

#### Data Analysis

The interview recordings were transcribed verbatim, translated into English, and then subjected to thematic analysis to identify positive and negative aspects of acceptability and practicality. Descriptive statistics were calculated to describe participants’ characteristics and changes in outcomes. Changes in outcomes from pre- to posttreatment were compared between the tele-Indo-DARPP+TAU and TAU-only arms using the Wilcoxon signed rank test. The statistical significance level was set at .05. Data analysis was conducted with Stata (version 17.0; StataCorp).

### Ethical Considerations

Potential participants were informed of the purpose of the study, methods, burdens, and expected risks and benefits of participation, the voluntary nature of consent, and that consent could be withdrawn at any time. Participation was only acknowledged if informed consent was obtained. All study participants were asked to sign a pledge regarding group therapy participation including the commitment to keep discussions confidential and to not share any information about other participants with any third party. This study protocol was approved by the ethics review committees of the Faculty of Medicine, Universitas Indonesia (approval number KET-1175/2019) and the Graduate School of Medicine, Kyoto University (approval number C1483).

## Results

### Study Participants

Among the 8 participants who participated in the study, 4 declared their preference for and were thus allocated to the tele-Indo-DARPP+TAU arm, with the remaining 4 participants being allocated to the TAU-only arm. One of the participants allocated to the TAU-only arm did not complete the posttreatment assessment due to lost contact; the participant was determined as “dropped out” and thus not included in the outcome analysis.

The demographics of the participants at pretreatment are shown in Table 2. The mean age was 37 (SD 12.8) years, and all participants were male. The majority of the participants had completed high school or higher education (7/8, 88%), had a part-time job (6/8, 75%), were widowed or separated or never married (6/8, 75%), lived in a household with 3 persons or more (6/8, 75%), were Muslims (7/8, 88%), and belonged to an ethnic group rooted in Java island (6/8, 75%). The mean time and cost needed for one-way transportation to the nearest health care facility were 80 (SD 39.6) minutes and 36,900 (SD 18,300) rupiah (US $1=14,760 rupiah), respectively. Half of the participants had been arrested due to drug charges. Sedatives were reported by 7 participants (87.5%) as their primary drug of concern, while 1 reported ATS. Sedatives, alcohol, and methadone were reported to have been used in the past 30 days.

**Table 2:** Participant demographics at pretreatment.

Demographics		Total (n=8)	Tele-Indo-DARPP+TAU^a^ (n=4)	TAU^b^-only (n=4)
Age (years), mean (SD)		37.0 (12.8)	39.3 (5.5)	34.8 (18.4)
Sex (male), n (%)		8 (100)	4 (100)	4 (100)
**Education completed, n (%)**				
	Junior high	1 (12)	1 (25)	0 (0)
	High	4 (50)	2 (50)	2 (50)
	Vocational	3 (38	1 (25)	2 (50)
**Employment status, past 3 months, n (%)**				
	Full-time job	1 (12)	1 (25)	0 (0)
	Part-time job	6 (75)	2 (50)	4 (100)
	Disabled	1 (12)	1 (25)	0 (0)
**Marital status, n (%)**				
	Married	2 (25)	1 (25)	1 (25)
	Widowed or separated	2 (25)	1 (25)	1 (25)
	Never married	4 (50)	2 (50)	2 (50)
**Household size, n (%)**				
	Living alone	0 (0)	0 (0)	0 (0)
	2 persons	2 (25)	0 (0)	2 (50)
	3 persons or more	6 (75)	4 (100)	2 (50)
**Religion, n (%)**				
	Islam	7 (88)	3 (75)	4 (100)
	Christianity	1 (12)	1 (25)	0 (0)
**Ethnicity, n (%)**				
	Jawa	3 (38)	1 (25)	2 (50)
	Sunda	2 (25)	0 (0)	2 (50)
	Betawi	1 (12)	1 (25)	0 (0)
	Minang	1 (12)	1 (25)	0 (0)
	East Nusa Tenggara	1 (12)	1 (25)	0 (0)
**Transportation to the nearest health care facility, one-way trip, mean (SD)**				
	Time in minutes	80 (39.6)	81.3 (37.5)	80 (46.9)
	Cost in thousands of rupiah (US $1=14,760 rupiah)	36.9 (18.3)	32.5 (20.6)	41.2 (17.5)
**Number of arrests due to drug charges, n (%)**				
	Never	4 (50)	1 (25)	3 (75)
	1-2 times	2 (25)	2 (50)	0 (0)
	3 times or more	2 (25)	1 (25)	1 (25)
**Primary drug of concern, n (%)**				
	Sedatives	7 (88)	4 (100)	3 (75)
	Amphetamines	1 (12)	0 (0)	1 (25)
**Drug type used in the past 30 days, n (%)**				
	Alcohol	3 (38)	2 (25)	1 (25)
	Heroin	0 (0)	0 (0)	0 (0)
	Methadone	2 (25)	1 (25)	1 (25)
	Other opioids	0 (0)	0 (0)	0 (0)
	Barbiturates	0 (0)	0 (0)	0 (0)
	Sedatives	8 (100)	4 (100)	4 (100)
	Cocaine	0 (0)	0 (0)	0 (0)
	Amphetamines	0 (0)	0 (0)	0 (0)
	Cannabis	0 (0)	0 (0)	0 (0)
	Inhalants	0 (0)	0 (0)	0 (0)

^a^tele-Indo-DARPP: tele-Indonesia Drug Addiction Relapse Prevention Program.

^b^TAU: treatment as usual.

### Indo-DARPP Session Attendance

Among the 4 participants allocated to the tele-Indo-DARPP+TAU arm, 2 participants joined all 12 tele-Indo-DARPP sessions, with 1 participant attending 11 sessions and the other attending 9 sessions. Collective attendance was 92% (44 sessions).

### Results of Feedback Interviews

The participants who joined the tele-Indo-DARPP reported that, in general, the module had a good acceptability in terms of being able to open up and disclose personal matters, receiving helpful counselor’s advice, learning new things, feeling support from other participants, and broadening their view by listening to and sharing with other participants at various stages of recovery from SUD. In contrast, participants expressed complaints regarding the tele-Indo-DARPP contents, finding that some of its terminologies were confusing and did not adhere to the actual colloquial terms commonly used in the streets (eg, nicknames for substances) and that some of the medical information was too complicated for lay people and not sufficiently accessible. The interview data are provided in Textbox 1.

In terms of practicality, the participants reported the convenience of joining from home, without having to waste any time or money on transportation, and felt that the pacing between sessions was good. However, participants sometimes felt frustrated with technical issues and unstable internet connection and wanted more time to share their stories. In the end, 75% (n=3) of participants expressed their preference for web telemedicine over face-to-face meetings.

No adverse effects of tele-Indo-DARPP were reported. We confirmed with participants that they did not perceive that tele-Indo-DARPP negatively affected any of their physical, psychological, or social conditions.

**Themes and illustrative interview raw data on the acceptability and practicality of tele-Indonesia Drug Addiction Relapse Prevention Program from the point of view of participants and service providers.**

**Acceptability: well accepted**

- Able to open up on personal matters: “Talking about my meth use reminded me of all the things, like my ex-girlfriend and my old friends who died of AIDS. I had never talked about it with anyone before. I never thought of sharing it because nobody would understand me anyway. But in the program, others also shared similar things and I felt accepted.”
- Found the advice from peer counselors relatable: “I learned the meaning of [taking] one day at a time from him [the peer counselor]. Even if I used drugs yesterday, it’s important to start another new day [from] there and try to live better at least on that day. Repeat and build on it. That sort of thinking helps a lot.”
- Learned new things from texts: “Making a schedule was new to me. Keeping myself busy can distract me from things I want to forget.”
- Felt supported by others: “I thought no one would care about me, but that’s not true when I was in the program. I could complete the program because everyone, I mean other clients too, supported me.”
- Learned mutually among clients: “It was very interesting to hear the various viewpoints from the others here, some of whom have already battled addiction for 20 years. I am eager to join future sessions so I can hear more and more stories from anyone [at] different stages in their path to recovery, including new clients, whom I can help by teaching my own experiences as well.”
- (Providers) module is provider-friendly: “As a psychiatrist, I am familiar with the concept and methodologies in CBT (cognitive behavioral therapy). However, for general practitioners and nurses who do not have CBT experience, by closely following this module and the workbook, the service has become exactly like CBT.”

**Acceptability: poorly accepted**

- Terminologies used in the workbook are not close enough to reality: “Sometimes the workbook content felt a bit distant from the reality in the streets. I hope there can be content revision to make us feel more familiar [with the workbook], such as adding brand names for prescription drugs, and street names for illicit drugs.”
- Explanations of medical and statistical information are not accessible enough: “Sometimes I couldn’t really understand the medical explanation or study graphs, and tend[ed] to just skim them.”
- (Providers) lengthy materials in the workbook: “We had to hurry because there was too much material for a 2-hour session. At the same time, I was afraid if the session became too long, the client may lose focus.”

**Practicality: practical**

- Convenient to join the session from home: “I really like that I could join it from home, even right after taking a nap. Going to the hospital is such a hassle.”
- No wasted time or cost for transportation: “If I go to the hospital, it will take up my half-day because of the terrible traffic jam and I need to take [time] off from my job.”
- Good pacing between sessions: “One session per week was the best frequency for me. I could remember what we learned in the last session.”
- (Providers) convenient to join while still making rapport with clients: “[Ever since the pandemic] I am already getting used to providing teleconference services. My concern was that I couldn’t make proper rapport with clients, but as it turns out, they seemed comfortable in sharing their experiences and opinions. Probably because they already felt comfortable by participating from their home; their own comfort zone.”

**Practicality: impractical**

- Frustrated with technical issues: “I could not use my video sometimes, which was so frustrating. I couldn’t keep up with the discussion because of the technical problems, and then I lost the motivation I had before.”
- Wanted more time to share: “I wanted more time to share. Because it’s a group session, time for each of us is more limited compared to one-on-one sessions.”
- (Providers) unstable connection: “One of the participants could not have a stable connection, and his voice kept getting interrupted; probably his house had a bad (telecommunication) signal. At that time, I asked him to turn off the video (to improve bandwidth) for a moment or write what he wanted to say in the chat box.”

### Outcome Changes

**Figure 2:**
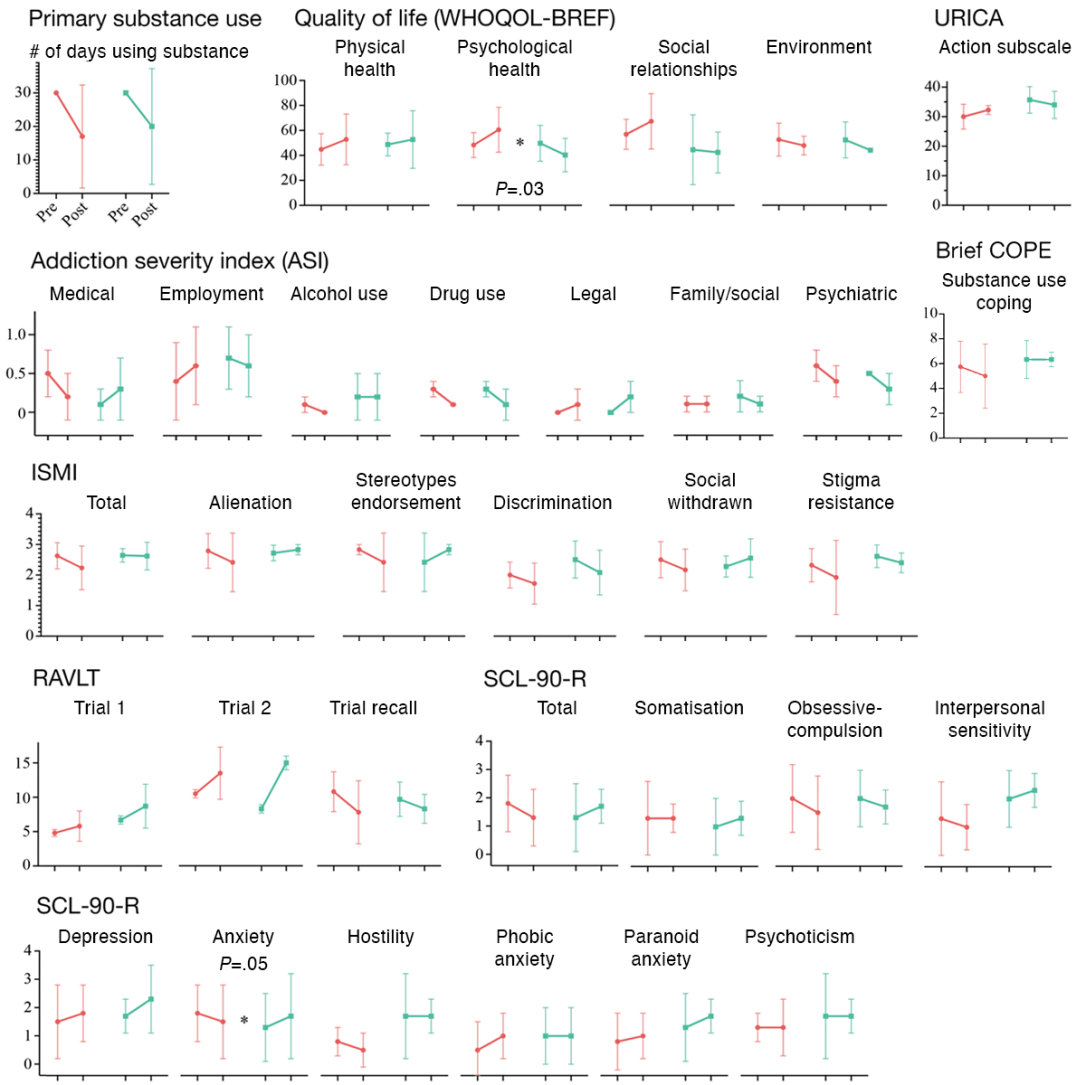
Differences between pre- and postintervention measurements of primary (primary substance use) and secondary outcomes (ASI, WHOQOL-BREF, URICA, Brief COPE, RAVLT, ISMI, and SCL-90-R scores) in the pilot study. The mean and SD (error bar) are shown for each measurement. Results of the tele-Indo-DARPP+TAU group are shown in red, and the TAU-only group in green (*P<.05). ASI: Addiction Severity Index; Brief COPE: Brief-Coping Orientation to Problems Experienced; ISMI: internalized stigma of mental illness; RAVLT: Rey Auditory Verbal Learning Test; SCL-90-R: Symptom Checklist-90 Revised; TAU: treatment as usual; tele-Indo-DARPP: tele-Indonesia Drug Addiction Relapse Prevention Program; URICA: University of Rhode Island Change Assessment; WHOQOL-BREF: World Health Organization Quality of Life Brief Version.

Figure 2 shows the mean and SD of the outcome measurements at pre- and posttreatment, stratified by the tele-Indo-DARPP+TAU arm and the TAU-only arm. The corresponding details can be found in Table S1 in Multimedia Appendix 1. The score in the psychological health domain of WHOQOL-BREF increased by a median of 12.5 points among the tele-Indo-DARPP+TAU arm, contrasting with the TAU-only arm whose scores decreased by a median of 8.3 (*P*=.03), also, the anxiety domain score of the Symptom Checklist-90 Revised showed a decrease with a median of 0.3 among the tele-Indo-DARPP+TAU arm participants, while that of the TAU-only group increased with a median of 0.2 (*P*=.05). We did not observe statistically significant differences in the other outcomes.

## Discussion

### Principal Findings

In studies testing tele-psychotherapies for SUD, tele-Indo-DARPP is only the second intervention formed as group therapy on a videoconferencing platform after a 2009 study [36] and is the first to be implemented in an LMIC. While all prior intervention modules only focused on a single substance (alcohol or opioids) [64], the tele-Indo-DARPP addresses 6 groups of substances: opioids, ATS, benzodiazepines, alcohol, marijuana, and NPS. Nevertheless, this pilot study did not recruit participants using various substances, and we hope to recruit more variable and representative participants in the RCT (protocol outlined by Yamada et al [49]) which we started several months after the conclusion of this pilot study and is now ongoing.

Outcome measurements showed that participants who received tele-Indo-DARPP+TAU exhibited improvements in their psychological health and anxiety symptoms, compared with those receiving TAU only. As shown in the interviews, participants were able to disclose private issues during the tele-Indo-DARPP sessions to the extent that they shared about their illegal activities and the bereavement of persons significant to them. It could be inferred that tele-Indo-DARPP nurtured a supportive group environment, which alleviated anxiety and enhanced psychological health. Such a finding is noteworthy given that previous studies have shown that online psychotherapies face difficulties in rapport building [65]. Building rapport has also been reported the most as a challenge in group therapy settings [66]. Our success in building a safe and private environment for clients in an SUD treatment setting may partly be owing to the involvement of peer counselors and the establishment of a common understanding of group rules as listed in Methods.

Our interview data showed that, in general, tele-Indo-DARPP had good acceptability and practicality. Tele-Indo-DARPP was perceived as time-efficient by the participants of our pilot study whose residence was in Jakarta, the world’s second-largest urban area, where traffic is inconvenient and heavily congested. Indeed, tele-Indo-DARPP could save over 2 hours of round-trip transportation time to visit an outpatient clinic. The increased time efficiency allowed participants to join tele-Indo-DARPP sessions weekly without having conflicting schedules for their jobs or childcare. In contrast, in a face-to-face TAU, SUD patients typically cannot visit clinics more than once a month due to time and cost restraints. This is important as the frequency of therapy attendance is known to improve health outcomes among SUD clients. This finding is in line with previous studies reporting that the top reasons for SUD outpatients preferring telehealth care included the ability to receive it from home and not needing to spend time commuting [36,66].

Nevertheless, the interview results revealed several issues regarding the practicality and acceptability of tele-Indo-DARPP. The participants reported that they were not familiar with some terminologies, such as the substance chemical names, and had difficulties in comprehending medical and statistical information. The workbook contents were carefully developed to increase comprehensibility, but further considerations are necessary due to the relatively low educational backgrounds of people with SUD, such as adding the colloquial street names of substances, explanatory pictures, and presenting complex knowledge more simply. Also, the participants felt frustrated when having technical issues related to unstable internet connections or smartphone devices, which is another obstacle to the widespread implementation of telemedicine.

### Strengths and Limitations

A key strength of this study is that this is the first trial of tele-psychotherapy using videoconferencing in an LMIC. This is also the first trial for tele-psychotherapy for SUD in Indonesia, where any standardized guideline for SUD psychotherapy is nonexistent. Indo-DARPP may become the first structured therapeutic module focusing on substance craving and relapse prevention in Indonesia. Moreover, it is the first trial of psychotherapy coprovision by a peer and a health care worker in Indonesia. We showed the feasibility of tele-psychotherapy and peer involvement in formal SUD care, which has not been well investigated not only in Indonesia but also in LMICs, and reported preliminary positive results of outcome changes following therapy.

As this pilot study is of an exploratory and preliminary nature, with a limited sample size, and was not intended to determine clinical effectiveness, the results should be interpreted with caution. Nonetheless, we observed significant improvements in psychological health and anxiety symptoms, as well as encouraging outcomes in the Addiction Severity Index medical domain, substance use coping, and several subscales related to internalized stigma, despite the lack of statistical significance. Further limitations were that the study participants were all male, most reported sedatives as their primary substance, and were recruited in urban areas in the capital city of Jakarta. Although being male and urban residency are predominant characteristics in people who use drugs in Indonesia [3], the observed acceptability, practicality, and preliminary outcome changes might not be generalizable to different populations in Indonesia. We hope to improve the demographic variability of participants and expand recruitment from many distinct islands nationwide in the future tele-Indo-DARPP RCT.

### Conclusions

The preliminary results of our pilot study gave some encouraging insight into how peer counselors can be involved in formal psychotherapeutic sessions for clients with SUD and how such therapy can be delivered via videoconferencing in Indonesia. Future effectiveness studies should explore the best recruitment place and source of providers for such community-based therapy, that is, whether it should be run by rehabilitation centers, Puskesmas, or hospitals. Currently, we are conducting a clinical trial recruiting participants in different outpatient settings [49]. Future implementation studies should also make sure to include diverse populations, especially in terms of gender and location within the vast archipelago of Indonesia.

## Appendix

Multimedia Appendix 1 Supplementary Figure 1 and Supplementary Table 1.

## Abbreviations

ATS: amphetamine-type stimulant
CBT: cognitive behavioral therapy
Indo-DARPP: Indonesia Drug Addiction Relapse Prevention Program
LMIC: low- and middle-income country
NPS: new psychoactive substance
RCT: randomized clinical trial
SMARPP: Serigaya Methamphetamine Relapse Prevention Program
SUD: substance use disorder
TAU: treatment as usual
tele-Indo-DARPP: tele-Indonesia Drug Addiction Relapse Prevention Program
WHOQOL-BREF: World Health Organization Quality of Life Brief Version
