# Supplementary material for: Relapse Prevention Group Therapy in Indonesia Involving Peers via Videoconferencing for Substance Use Disorder: Development and Feasibility Study
Source: JMIR Form Res. 2024 Jun 18;8:e50452. doi: 10.2196/50452 (PMC11220436; doi:10.2196/50452)
Supplement: Multimedia Appendix 1 [file formative_v8i1e50452_app1.pdf]

1 (a)

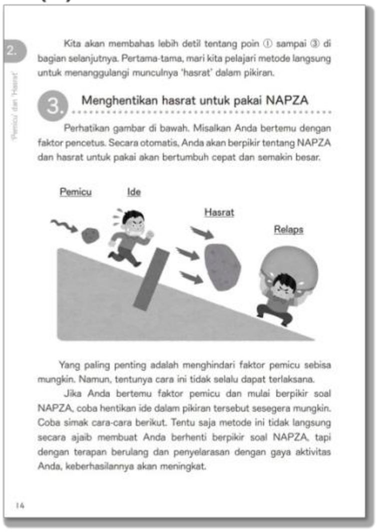

2. 'Pemicu' dan 'Hasrat'

Kita akan membahas lebih detil tentang poin ① sampai ③ di bagian selanjutnya. Pertama-tama, mari kita pelajari metode langsung untuk menanggulangi munculnya 'hasrat' dalam pikiran.

3. Menghentikan hasrat untuk pakai NAPZA

Perhatikan gambar di bawah. Misalkan Anda bertemu dengan faktor pencetus. Secara otomatis, Anda akan berpikir tentang NAPZA dan hasrat untuk pakai akan bertumbuh cepat dan semakin besar.

Yang paling penting adalah menghindari faktor pemicu sebisa mungkin. Namun, tentunya cara ini tidak selalu dapat terlaksana.

Jika Anda bertemu faktor pemicu dan mulai berpikir soal NAPZA, coba hentikan ide dalam pikiran tersebut sesegera mungkin. Coba simak cara-cara berikut. Tentu saja metode ini tidak langsung secara ajaib membuat Anda berhenti berpikir soal NAPZA, tapi dengan terapan berulang dan penyelarasan dengan gaya aktivitas Anda, keberhasilannya akan meningkat.

14

1 (b)

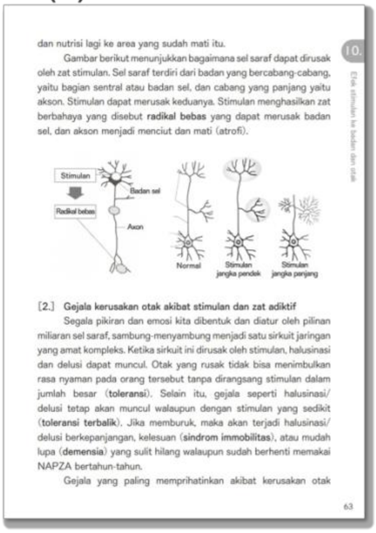

dan nutrisi lagi ke area yang sudah mati itu.

Gambar berikut menunjukkan bagaimana sel saraf dapat dirusak oleh zat stimulan. Sel saraf terdiri dari badan yang bercabang-cabang, yaitu bagian sentral atau badan sel, dan cabang yang panjang yaitu akson. Stimulan dapat merusak keduanya. Stimulan menghasilkan zat berbahaya yang disebut **radikal bebas** yang dapat merusak badan sel, dan akson menjadi menciut dan mati (atrofi).

[2.] **Gejala kerusakan otak akibat stimulan dan zat adiktif**

Segala pikiran dan emosi kita dibentuk dan diatur oleh pilinan miliaran sel saraf, sambung-menyambung menjadi satu sirkuit jaringan yang amat kompleks. Ketika sirkuit ini dirusak oleh stimulan, halusinasi dan delusi dapat muncul. Otak yang rusak tidak bisa menimbulkan rasa nyaman pada orang tersebut tanpa dirangsang stimulan dalam jumlah besar (**toleransi**). Selain itu, gejala seperti halusinasi/delusi tetap akan muncul walaupun dengan stimulan yang sedikit (**toleransi terbalik**). Jika memburuk, maka akan terjadi halusinasi/delusi berkepanjangan, kelesuan (**sindrom immobilitas**), atau mudah lupa (**demensia**) yang sulit hilang walaupun sudah berhenti memakai NAPZA bertahun-tahun.

Gejala yang paling memprihatinkan akibat kerusakan otak

10. Efek stimulan ke badan dan otak

63

1 (c)

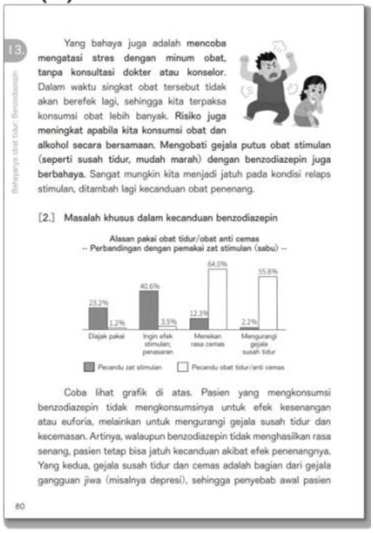

13. Bahayanya obat tidur: Benzodiazepin

Yang bahaya juga adalah **mencoba mengatasi stres dengan minum obat, tanpa konsultasi dokter atau konselor.** Dalam waktu singkat obat tersebut tidak akan berefek lagi, sehingga kita terpaksa konsumsi obat lebih banyak. **Risiko juga meningkat apabila kita konsumsi obat dan alkohol secara bersamaan. Mengobati gejala putus obat stimulan (seperti susah tidur, mudah marah) dengan benzodiazepin juga berbahaya.** Sangat mungkin kita menjadi jatuh pada kondisi relaps stimulan, ditambah lagi kecanduan obat penenang.

[2.] **Masalah khusus dalam kecanduan benzodiazepin**

Coba lihat grafik di atas. Pasien yang mengkonsumsi benzodiazepin tidak mengkonsumsinya untuk efek kesenangan atau euforia, melainkan untuk mengurangi gejala susah tidur dan kecemasan. Artinya, walaupun benzodiazepin tidak menghasilkan rasa senang, pasien tetap bisa jatuh kecanduan akibat efek penenangnya. Yang kedua, gejala susah tidur dan cemas adalah bagian dari gejala gangguan jiwa (misalnya depresi), sehingga penyebab awal pasien

80

1 (d)

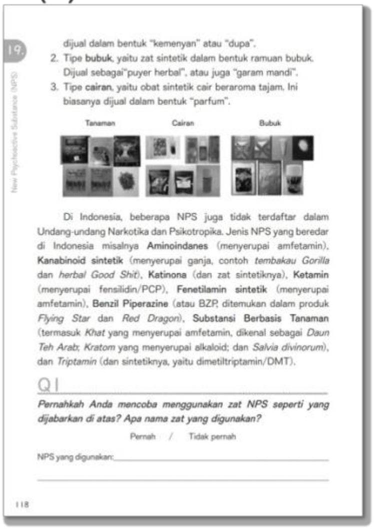

19. New Psychoactive Substance (NPS)

dijual dalam bentuk "kemenyan" atau "dupa".

2. Tipe **bubuk**, yaitu zat sintetik dalam bentuk ramuan bubuk. Dijual sebagai"puyer herbal", atau juga "garam mandi".
3. Tipe **cairan**, yaitu obat sintetik cair beraroma tajam. Ini biasanya dijual dalam bentuk "parfum".

Di Indonesia, beberapa NPS juga tidak terdaftar dalam Undang-undang Narkotika dan Psikotropika. Jenis NPS yang beredar di Indonesia misalnya **Aminoindanes** (menyerupai amfetamin), **Kanabinoid sintetik** (menyerupai ganja, contoh *tembakau Gorilla* dan *herbal Good Shit*), **Katinona** (dan zat sintetiknya), **Ketamin** (menyerupai fensilidin/PCP), **Fenetilamin sintetik** (menyerupai amfetamin), **Benzil Piperazine** (atau BZP, ditemukan dalam produk *Flying Star* dan *Red Dragon*), **Substansi Berbasis Tanaman** (termasuk *Khat* yang menyerupai amfetamin, dikenal sebagai *Daun Teh Arab*; *Kratom* yang menyerupai alkaloid; dan *Salvia divinorum*), dan *Triptamin* (dan sintetiknya, yaitu dimetiltriptamin/DMT).

Q1

Pernahkah Anda mencoba menggunakan zat NPS seperti yang dijabarkan di atas? Apa nama zat yang digunakan?

Pernah / Tidak pernah

NPS yang digunakan:____________________

118

2 (a)

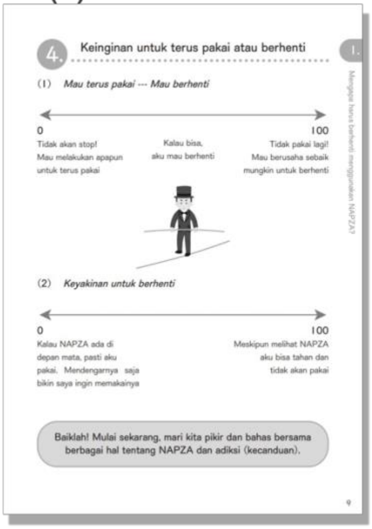

4. **Keinginan untuk terus pakai atau berhenti**

(1) ***Mau terus pakai --- Mau berhenti***

0 — 100

Tidak akan stop! Mau melakukan apapun untuk terus pakai

Kalau bisa, aku mau berhenti

Tidak pakai lagi! Mau berusaha sebaik mungkin untuk berhenti

(2) ***Keyakinan untuk berhenti***

0 — 100

Kalau NAPZA ada di depan mata, pasti aku pakai. Mendengarnya saja bikin saya ingin memakainya

Meskipun melihat NAPZA aku bisa tahan dan tidak akan pakai

Baiklah! Mulai sekarang, mari kita pikir dan bahas bersama berbagai hal tentang NAPZA dan adiksi (kecanduan).

1. Mengapa harus berhenti menggunakan NAPZA?

9

2 (b)

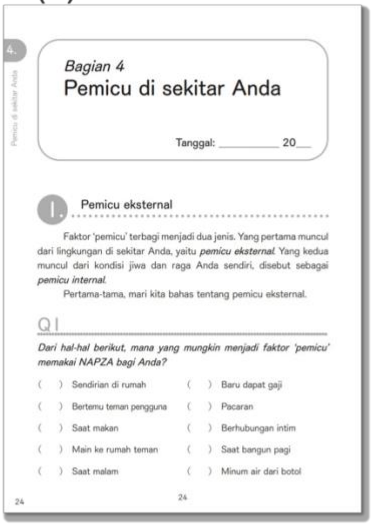

4. Pemicu di sekitar Anda

Bagian 4

Pemicu di sekitar Anda

Tanggal: __________ 20__

1. **Pemicu eksternal**

Faktor 'pemicu' terbagi menjadi dua jenis. Yang pertama muncul dari lingkungan di sekitar Anda, yaitu ***pemicu eksternal***. Yang kedua muncul dari kondisi jiwa dan raga Anda sendiri, disebut sebagai ***pemicu internal***.

Pertama-tama, mari kita bahas tentang pemicu eksternal.

Q1

Dari hal-hal berikut, mana yang mungkin menjadi faktor 'pemicu' memakai NAPZA bagi Anda?

( ) Sendirian di rumah	( ) Baru dapat gaji
( ) Bertemu teman pengguna	( ) Pacaran
( ) Saat makan	( ) Berhubungan intim
( ) Main ke rumah teman	( ) Saat bangun pagi
( ) Saat malam	( ) Minum air dari botol

24

24

4 (a)

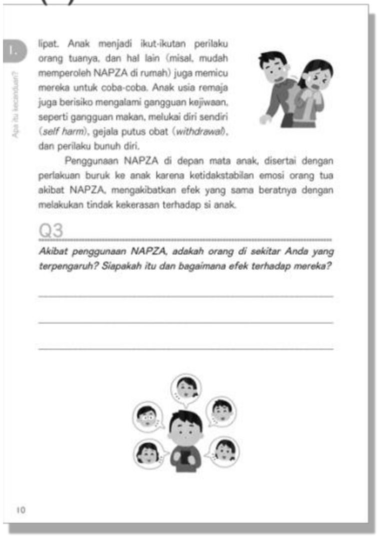

1. Apa itu kecanduan?

lipat. Anak menjadi ikut-ikutan perilaku orang tuanya, dan hal lain (misal, mudah memperoleh NAPZA di rumah) juga memicu mereka untuk coba-coba. Anak usia remaja juga berisiko mengalami gangguan kejiwaan, seperti gangguan makan, melukai diri sendiri (*self harm*), gejala putus obat (*withdrawal*), dan perilaku bunuh diri.

Penggunaan NAPZA di depan mata anak, disertai dengan perlakuan buruk ke anak karena ketidakstabilan emosi orang tua akibat NAPZA, mengakibatkan efek yang sama beratnya dengan melakukan tindak kekerasan terhadap si anak.

Q3

Akibat penggunaan NAPZA, adakah orang di sekitar Anda yang terpengaruh? Siapakah itu dan bagaimana efek terhadap mereka?

10

4 (b)

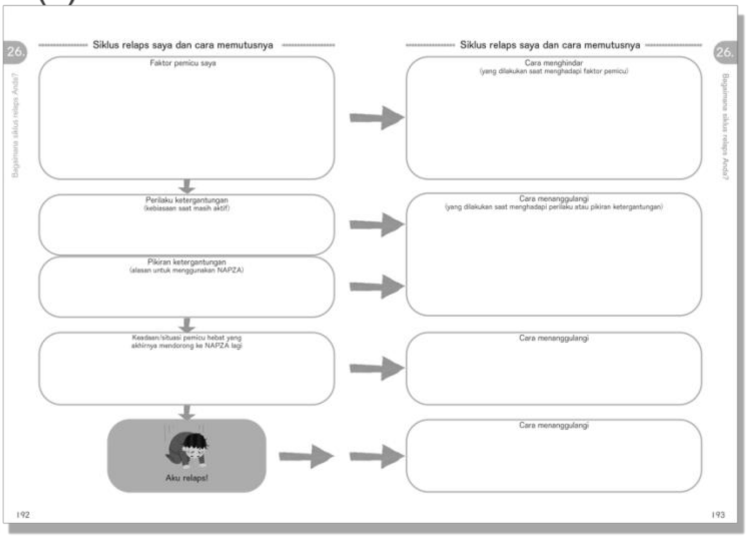

26. Bagaimana siklus relaps Anda?

Siklus relaps saya dan cara memutusnya

Faktor pemicu saya → Cara menghindar (yang dilakukan saat menghadapi faktor pemicu)

Perilaku ketergantungan (kebiasaan saat masih aktif) → Cara menanggulangi (yang dilakukan saat menghadapi perilaku atau pikiran ketergantungan)

Pikiran ketergantungan (alasan untuk menggunakan NAPZA) → Cara menanggulangi (yang dilakukan saat menghadapi perilaku atau pikiran ketergantungan)

Keadaan/situasi pemicu hebat yang akhirnya mendorong ke NAPZA lagi → Cara menanggulangi

Aku relaps! → Cara menanggulangi

192 193

Figure S1. Sample pages of the Indo-DARPP workbook, showing various elements of the text. Brief explanation of each page are as follows.

1. User information:
(a) basic information, explaining concepts of trigger, craving, and relapse in a simple figure.
(b) medical information, detailing how stimulant may affect and damage the neurons.
(c) evidence-based data, showing discrepancy in reasons to use stimulant (grey bar) or sedatives (white bar), showed in percentages of respondents in a study.
(d) practical facts, listing types and examples of new psychoactive substances commonly found in the Indonesian market.

2. Self-assessment queries, which are specific or multiple-choice questions to invite readers in recognizing their own situations:
(a) Specific question regarding motivation to continue or stop using substances, presented in a 0-100 score line for readers to approximate their own ambivalence.
(b) Multiple-choice question regarding potential trigger to use substances.

3. Motivational interview, which are open-ended questions to help readers analyze themselves deeper and explore toward a solution and increased motivation:
(a) Open-ended question regarding reader's opinion on the impact of using substances toward the people around them, followed by specific question on who they were and how were they affected.
(b) Schematic showing the relapse cycle. Questions ask past and future or potential triggers, addiction-related behavior and thoughts, immediate triggers, and steps to anticipate and overcome them. This cycle is shown at the end of the book and used as a closure of the 12-week therapy. It is hoped that participants can come back to this analysis whenever they encounter risk of relapse in the future.

Table S1. Comparison of changes in outcomes between tele-Indo-DARPP + TAU and TAU only

		Tele-Indo-DARPP + TAU					TAU only					*P*	
		n = 4					n = 3						
		Mean	(SD)	Median	(Q1,	Q3)	Mean	(SD)	Median	(Q1,	Q3)		
Number of days using primary drug													
		-13.0	(15.4)	-26.0	(-30.0 ,	-22.0)	-10.0	(17.3)	0.0	(-30.0 ,	0.0)	0.361	
ASI													
	Medical	-0.3	(0.3)	-0.2	(-0.5 ,	-0.0)	0.1	(0.5)	0.0	(-0.3 ,	0.7)	0.212	
	Employment	0.2	(0.5)	0.0	(-0.0 ,	0.5)	-0.1	(0.2)	0.0	(-0.4 ,	0.0)	0.271	
	Alcohol use	-0.1	(0.1)	0.0	(-0.1 ,	0.0)	0.0	(0.1)	0.0	(-0.1 ,	0.2)	0.593	
	Drug use	-0.2	(0.1)	-0.2	(-0.2 ,	-0.1)	-0.1	(0.1)	-0.1	(-0.2 ,	0.0)	0.724	
	Legal	0.1	(0.2)	0.0	(0.0 ,	0.2)	0.2	(0.2)	0.2	(0.0 ,	0.4)	0.430	
	Family/ social	0.0	(0.1)	0.0	(0.0 ,	0.1)	-0.1	(0.1)	0.0	(-0.1 ,	0.0)	0.079	
	Psychiatric status	-0.1	(0.2)	-0.2	(-0.3 ,	-0.0)	-0.2	(0.2)	-0.3	(-0.3 ,	0.0)	0.480	
WHOQOL-BREF													
	Physical health	8.0	(13.0)	5.4	(-1.8 ,	17.9)	4.0	(15.7)	-3.6	(-7.1 ,	21.4)	0.480	
	Psychological health	12.3	(9.5)	12.5	(4.2 ,	20.8)	-9.3	(2.3)	-8.3	(-12.5 ,	-8.3)	0.031	*
	Social relationships	12.5	(14.2)	8.3	(4.2 ,	20.8)	-2.7	(19.3)	8.3	(-25.0 ,	8.3)	0.593	
	Environment	-4.8	(11.6)	0.0	(-10.9 ,	1.6)	-8.3	(14.4)	0.0	(-25.0 ,	0.0)	0.435	
URICA													
	Action	2.3	(5.7)	2.5	(-2.5 ,	7.0)	-1.7	(5.5)	-2.0	(-7.0 ,	4.0)	0.289	
Brief-COPE													
	Substance use coping	-0.8	(1.5)	-1.0	(-2.0 ,	0.5)	0.0	(2.0)	0.0	(-2.0 ,	2.0)	0.578	
SCL-90-R													
	GSI	-0.5	(0.6)	-0.1	(-0.3 ,	0.1)	0.3	(0.6)	-0.1	(-0.1 ,	0.3)	0.480	
	Somatisation	0.0	(0.8)	0.2	(-0.4 ,	0.4)	0.3	(0.6)	-0.3	(-0.3 ,	0.3)	0.724	

	Obsessive-Compulsion	-0.5	(0.6)	-0.3	(-0.4 , -0.1)	-0.3	(1.2)	-0.5	(-0.6 , 0.9)	0.593	
	Interpersonal sensitivity	-0.3	(0.5)	-0.1	(-0.6 , 0.1)	0.3	(0.6)	0.1	(-0.1 , 0.8)	0.212	
	Depression	0.3	(0.5)	0.1	(0.0 , 0.3)	0.7	(0.6)	0.3	(0.1 , 0.8)	0.480	
	Anxiety	-0.3	(0.5)	-0.3	(-0.5 , -0.1)	0.3	(0.6)	0.2	(0.0 , 0.7)	0.049	*
	Hostility	-0.3	(0.5)	-0.2	(-0.3 , -0.1)	0.0	(1.0)	0.3	(-1.0 , 0.5)	0.480	
	Phobic anxiety	0.5	(0.6)	-0.2	(-0.3 , -0.1)	0.0	(0.0)	0.3	(-1.0 , 0.5)	0.480	
	Paranoid ideation	0.3	(0.5)	-0.1	(-0.3 , 0.3)	0.3	(0.6)	0.0	(-0.5 , 0.7)	0.858	
	Psychoticism	0.0	(0.8)	-0.2	(-0.4 , 0.1)	0.0	(1.0)	-0.5	(-0.9 , 0.4)	0.480	
RAVLT											
	Trial 1	1.0	(2.2)	0.5	(0.0 , 2.5)	2.0	(2.6)	3.0	(-1.0 , 4.0)	0.857	
	Trial 2	3.0	(4.2)	0.0	(-2.0 , 1.5)	6.7	(0.6)	1.0	(1.0 , 2.0)	0.354	
	Recall	-3.0	(5.0)	-0.5	(-2.0 , 0.5)	-1.3	(4.5)	-1.0	(-6.0 , 3.0)	0.858	
ISMI											
	Total	-0.4	(1.0)	0.1	(-1.0 , 0.2)	0.0	(0.3)	0.0	(-0.4 , 0.3)	0.724	
	Alienation	-0.4	(1.0)	0.0	(-1.0 , 0.3)	0.1	(0.2)	0.0	(0.0 , 0.3)	0.721	
	Stereotype endorsement	-0.6	(1.1)	-0.4	(-1.4 , 0.1)	0.0	(0.6)	0.1	(-0.6 , 0.6)	0.373	
	Discrimination experience	-0.35	(1.0)	0.0	(-0.9 , 0.2)	-0.5	(0.5)	-0.6	(-1.0 , 0.0)	0.463	
	Social withdrawal	-0.3	(1.0)	0.2	(-0.8 , 0.2)	0.3	(0.7)	0.2	(-0.3 , 1.0)	1.000	
	Stigma resistance	-0.3	(1.1)	0.1	(-0.9 , 0.4)	-0.1	(0.1)	-0.2	(-0.2 , -0.2)	0.368	

ASI, Addiction Severity Index; WHOQOL-BREF, World Health Organization Quality of Life Brief Version; URICA, University of Rhode Island Change Assessment; Brief COPE, Brief-Coping Orientation to Problems Experienced; SCL-90-R, Symptom Checklist-90 Revised; GSI, Global Severity Index; RAVLT, Rey Auditory Verbal Learning Test; ISMI, Internalized Stigma of Mental Illness.
SD, standard deviation; Q1, the 1st quartile; Q3 the 3rd quartile; Indo-DARPP, Indonesia Drug Addiction Relapse Prevention Program; TAU, treatment as usual.
P values are for Wilcoxon's signed rank test. $^{*}P < 0.05$.
